# Diagnosis of Malaria Infection with or without Disease

**DOI:** 10.4084/MJHID.2012.036

**Published:** 2012-05-09

**Authors:** Zeno Bisoffi, Federico Gobbi, Dora Buonfrate, Jef Van den Ende

**Affiliations:** Centre for Tropical Diseases, Ospedale Sacro cuore, Negrar (Verona), Italy; 1 Department of Clinical Sciences, Institute of Tropical Medicine, Antwerp, Belgium

## Abstract

The revised W.H.O. guidelines for malaria management in endemic countries recommend that treatment should be reserved to laboratory confirmed cases, both for adults and children. Currently the most widely used tools are rapid diagnostic tests (RDTs), that are accurate and reliable in diagnosing malaria infection. However, an infection is not necessarily a clinical malaria, and RDTs may give positive results in febrile patients who have another cause of fever. Excessive reliance on RDTs may cause overlooking potentially severe non malarial febrile illnesses (NMFI) in these cases. In countries or areas where transmission intensity remains very high, fever management in children (especially in the rainy season) should probably remain presumptive, as a test-based management may not be safe, nor cost effective. In contrast, in countries with low transmission, including those targeted for malaria elimination, RDTs are a key resource to limit unnecessary antimalarial prescription and to identify pockets of infected individuals. Research should focus on very sensitive tools for infection on one side, and on improved tools for clinical management on the other, including biomarkers of clinical malaria and/or of alternative causes of fever.

## Background

Malaria management policies in most sub-Saharan countries traditionally relied on presumptive diagnosis without laboratory confirmation, basically considering any fever as malaria, particularly in children, being the most vulnerable to acute malaria.^1^ This was basically due to the lack of laboratory facilities at the peripheral level of the health system (Primary Health Care or P.H.C.). The diagnosis was then barely clinical, and while over treatment was an obvious consequence, the rationale behind such empirical management was the need to avoid missing possible malaria cases, especially in children, with potentially fatal consequences. Moreover, the treatment used in most countries until the beginning of the new millennium was chloroquine, a cheap and affordable drug, albeit increasingly ineffective against *Plasmodium falciparum* due to the spread of selected resistant strains. During the last decade, the new antimalarial Artemisinin Combined Treatments (ACT), that are extraordinarily effective but also orders of magnitude more expensive, have become the first choice for *P. falciparum* malaria almost everywhere.^2^ Presumptive treatment of all fevers with ACT would no more seem a logical and affordable choice, not only for economic reasons, but also because it is feared that a massive use of the new drugs might induce selection and diffusion of artemisinin-resistant strains of *P. falciparum*. For this reason the WHO policy changed, recommending restricting antimalarial treatment to laboratory confirmed cases.^2^

Unfortunately, the quality of malaria microscopy is far from satisfactory in most countries, especially in sub-Saharan Africa where most of malaria burden is concentrated. Moreover, the availability of microscopy is generally restricted to hospitals and private laboratories, while health centres and dispensaries generally lack any laboratory facility.

Rapid Diagnostic Tests (RDT) for malaria, based on antigen detection, were the potential solution. RDTs cannot be considered a new tool any more. The first tests, based on the detection of histidine rich protein (HRP-2) antigen of *P. falciparum*, were already commercially available at the beginning of the nineties,^3^ though much too expensive to be introduced in low to middle income countries (LMIC). They proved to be highly sensitive and specific, though with some false positives, especially in patients with a positive rheumathoid arthritis test.^4^ In following years, many new tests were developed, based either on HRP-2 or on lactic dehydrogenase (p-LDH), some of them restricted to *P. falciparum*, others capable of detecting specific antigens of the other malaria species, too. Initially developed as a potential tool for travellers to malaria endemic countries, the new tools were then progressively introduced and tested in the field, while their cost tended to diminish in parallel with an increased production and diffusion. RDTs were submitted to evaluation in many malarious countries,^5^–^10^ and were generally found to be accurate and cost – effective if compared to the previous, presumptive malaria diagnosis. The W.H.O. then recommended that, if and where microscopy was not available, RDTs should be obligatorily used in case of suspected malaria, and treatment should be restricted to positives.^2^ Initially, the new policy was indicated for older (> 5 years) chidren and adults, while the presumptive policy remained the recommended one for small children, on ground of a higher vulnerability to malaria. This empirical approach appeared reasonable. Missing a malaria diagnosis in an infant might be fatal, while this is less so, at least in high transmission settings, for older children and adults. Moreover, as the ACT dosage is related to the patient weight, the treatment of an adult is considerably more expensive than that of a child, and is generally higher than the cost of a RDT, too, while this is not necessarily true for the paediatric dosage.

## Malaria infection versus malaria disease

Infection does not necessarily mean disease, and this is true for many different etiologies and potential pathogens: viruses, bacteria, fungi, protozoa and helminths. As far as malaria is concerned, the infection may be characterised, for example, by the persistence of *P. vivax* or *P. ovale* “hypnozoites” in the liver, where they remain totally harmless for months or even years, until they eventually cause a relapse, reaching again the erythrocytes.^11^ For *P. falciparum* the liver stage is limited to the initial invasion, and the hypnozoite phenomenon does not occur. *P. falciparum* infection means the presence of trophozoites and schizonts in blood, where they undergo an asexual replication in RBCs, eventually destroying them when the schizonts rupture, liberating new merozoites which will infect new RBCs. The crucial event is the schizont (including the host RBC) rupture with the following delivery of substances both of the host RBC and of the plasmodium, which trigger cascades, largely mediated by several cytokines, responsible for the fever and the other main pathological aspects of malaria.^12^ The intensity of the erythrocitic cycle depends on several factors, the crucial one being the level of immunity of the infected subject which is related to previous exposure (the so called “premunition”).^13^ In areas with high intensity of transmission, acute and potentially fatal attacks are typical of infants and young children, while older children and adults are at least partially protected by premunition. They are still exposed to malaria, but acute attacks are less frequent and less severe. Moreover, a high proportion of apparently healthy subjects are carriers of malaria parasites in blood.

What is clinical malaria? How to define malaria disease as opposed to simple malaria infection? The most honest answer to this simple question would be, surprisingly enough: we don’t know.

## Malaria disease: fever plus plasmodia in blood?

For practical purposes, one could argue that clinical malaria is a fever (alone or with various combinations of other symptoms and signs) with presence of plasmodia in blood. Unfortunately, what is simple is not always true.

In a rural health centre of Banfora province, in Burkina Faso, during the dry season, a 2-year-old boy is admitted because of high fever, polypnoea and a visible, general prostration. The nurse in charge acts quickly: a malaria RDT is immediately performed and the result is positive. The child is immediately put on intravenous quinine drip. Unfortunately, the situation worsens in a few hours and the child dies the following morning.

Was there anything wrong in the case management? The nurse strictly followed the local guidelines, and i.v. artesunate was not yet available at the time. Clearly, a child may die of severe malaria, even if the management is appropriate, as in our case. But are we really sure that the child actually had malaria?

In endemic countries, the proportion of children who are carriers of malaria parasites in blood is variable, depending on the transmission intensity and the season, and may well be over 50% in some situations.^14^,^15^ This means that a child (or an adult) may have malaria infection despite the absence of any obvious symptom of disease, including fever. Suppose now that this child gets a pneumonia, or a typhoid, or a meningitis: what happens to malaria parasites? Will they disappear from the blood? Certainly not, although a transient decrease in parasite density may occur.^16^ This child will have fever plus malaria parasites in blood, and if submitted to a malaria test the result will be positive (and a true positive for that matter): yet, this child will NOT have clinical malaria, and if I am guided by the test result I can make a fatal mistake.

Then, is malaria disease a fever with malaria infection at high parasite density? The parasite density is the number of malaria parasites in the circulation, which is usually expressed per μL (or as percentage of infected RBC) and is usually estimated by microscopy by examining the thick film until we have counted 200 WBC, and all the infected RBC that we detect in the same microscopic fields.^17^ This is a rough estimate of course, moreover in severe infection schizonts tend to be sequestrated in the spleen, the liver, the brain and other internal organs and therefore a very high parasite burden may not be mirrored in the peripheral circulation.

It is certainly true that patients with clinical malaria tend to have a higher parasite density than subjects with a simple malaria infection, and there is no doubt that a patient with high fever and a 5% parasite density (or, say, >200,000 per μL) most probably HAS a clinical malaria. But what about a patient with high fever and a lower parasite density? May I exclude clinical malaria? Certainly not. This patient might also have clinical malaria, OR he may be a simple carrier of malaria parasites with another actual cause of fever. How do I know?

## Malaria disease: fever plus plasmodia in blood plus other typical symptoms?

Researchers have struggled to find clinical signs and symptoms that are good predictors or excluders of clinical malaria. Unfortunately, the results have been frustrating. Some symptoms such as vomiting may increase the probability of malaria in children, while other symptoms like cough may decrease that probability, but no symptom combination can actually confirm or rule out the suspicion of malaria in a febrile child.^18^

## Malaria attributable fever: an epidemiological concept

Researchers assessing the efficacy of malaria vaccines were confronted with the lack of a case definition of clinical malaria. In order to define the vaccine efficacy, simple malaria infection is not a good indicator: pragmatically, they are not really interested in how many infections are prevented by a vaccine, but rather in how many disease episodes (and related deaths) are potentially avoided by vaccination. Therefore, in order to find a proxy of clinical malaria, the epidemiologists involved in vaccine trials focused on fever, and attempted at estimating, in a given population, what proportion of fevers could be attributed to malaria.^16^ The basic concept was the well known epidemiological concept of the attributable risk (AR), or attributable fraction (AF), that aims at estimating, in a longitudinal or in a case-control study, how many cases of a given disease are attributable to one or more risk factors. The formulas are well known and may be retrieved in any epidemiological textbook.^19^ We briefly summarize how this is done starting from longitudinal data, that is, from two populations of infected and not infected children and assessing the proportion of fevers occurring in a short time in the two groups.^20^ If we call **f_p_** the proportion of febrile amongst parasitaemic children, and **f_p0_** the proportion of febrile children amongst aparasitaemiac children, then the **AF** (or the proportion of fevers attributable to malaria) will be:

$$\text{AF}=\left({\text{f}}_{\text{p}}-{\text{f}}_{\text{p0}}\right)/{\text{f}}_{\text{p}}$$

The following example (**Table 1**) is taken from a population study in children in the Gambia.^21^ More parasitaemic than aparasitaemic children have fever (41/127 or 32% versus 33/280 or 11%). But among the 41 febrile patients with malaria parasites in blood, the fever is not caused by malaria in all, as fever is also found in a (albeit smaller) proportion of uninfected children. The proportion of fevers that are attributable to malaria is represented by the AF.

$$\begin{array}{l} {\text{f}}_{\text{p}}=0.32\;\text{or}\;32\%\\ {\text{f}}_{\text{p0}}=0.11\;\text{or}\;11\%.\\ \text{AF}=\left(0.32-0.11\right)/0.32=0.63. \end{array}$$

**Figure 1.**
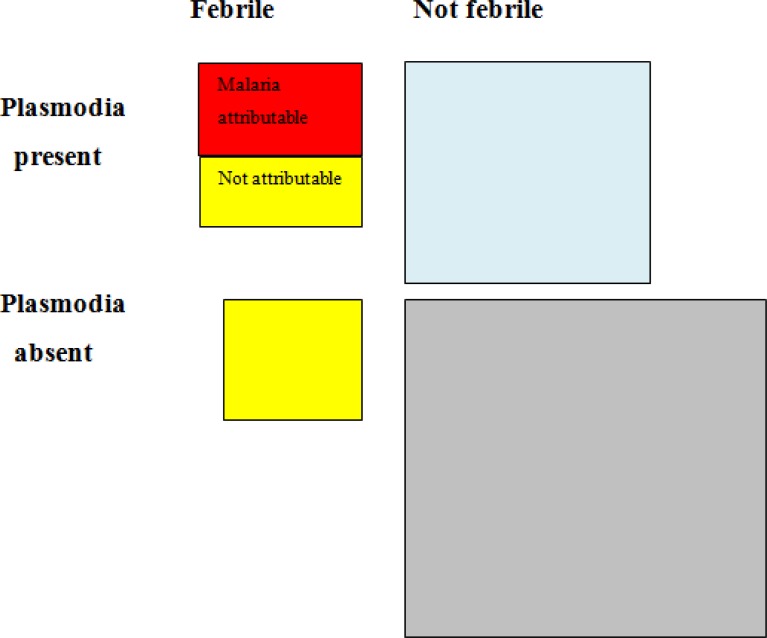
Grafic representation of the attributable fraction (AF) of fever to malaria infection, based on data in Table 1. The area of the squares is proportional to the size of each group. Fevers attributable to malaria (in red) are only a proportion of infected subjects with fever, while in yellow are represented fevers likely to be due to another cause, albeit some are observed in plasmodia carriers.

In normal language this means that 63% of children with fever and parasites are presumed to be ill due to malaria, in the other 37% the fever is probably caused by another disease (see **Figure 1** for a visual representation).

In a given endemic area, only a proportion of fevers among infected subjects is attributable to malaria. This proportion increases with parasite density, and over a given cut-off, virtually all fevers are attributable to malaria. The trouble is that this proportion is variable and depends on several factors, including the intensity of transmission, usually influenced by season and often variable across the years, and on the age of the subjects. Moreover, the AF is an epidemiological concept that cannot be automatically translated into individual diagnosis. In a rural area of Burkina Faso, we estimated that in the rainy season, among children > 5 years with fever and with a *P. falciparum* parasite density between 400 and 4000/μL, about half cases were attributable to malaria.^22^ If I see an individual patient with fever, and with a positive malaria film for *P.falciparum*, with parasite density say 2000/μL, what I can say is that my patient is about 50% likely to have an acute malaria attack, and 50% likely to be a carrier of malaria parasites in blood with another cause of his current fever.

## Malaria infection without disease: plasmodia in blood without fever?

Is asymptomatic malaria infection a harmless condition? Can we define infected subjects without fever as healthy carriers? The answer is no. Unfortunately, not much research has been done on the pathological effects of the long term presence of malaria infection in blood, but a strong epidemiological evidence exists that malaria infection produces pathological effects even in the absence of fever. Anemia is certainly more frequent/severe in children with malaria parasites in blood, and so is splenomegaly,^23^ to such an extent that the so called “spleen rate” is a marker of malaria prevalence that is almost as accurate as a lab based survey.^24^ Anemia and a big spleen are the most common markers of a “chronic malaria”, and these subjects cannot be defined as “healthy”. The most severe form of chronic malaria is hyper-reactive malarial splenomegaly (HMS), a neglected condition characterized by gross splenomegaly, pancytopenia, and a severely impaired cellular immunity.^25^ Plasmodia are present in blood, but at such a low parasite density that they may be missed, both by microscopy and by RDTs. This condition is invariably fatal if not adequately treated, and is probably the tip of the iceberg of the pathologic manifestations of a chronic malaria infection. A striking, indirect epidemiological indication of the harmful effects of malaria infection is the spectacular reduction of child mortality observed by some of the first trials of impregnated bed nets:^26^ the prevention of specific malaria mortality explained only a fraction of this reduction, indicating that malaria infection is a predisposing factor to death from other causes. Therefore, malaria infection is likely to produce pathological effects even in the absence of fever, and when it is detected, it should probably be treated.

## Diagnosis of malaria infection and disease

**Figure 2.**
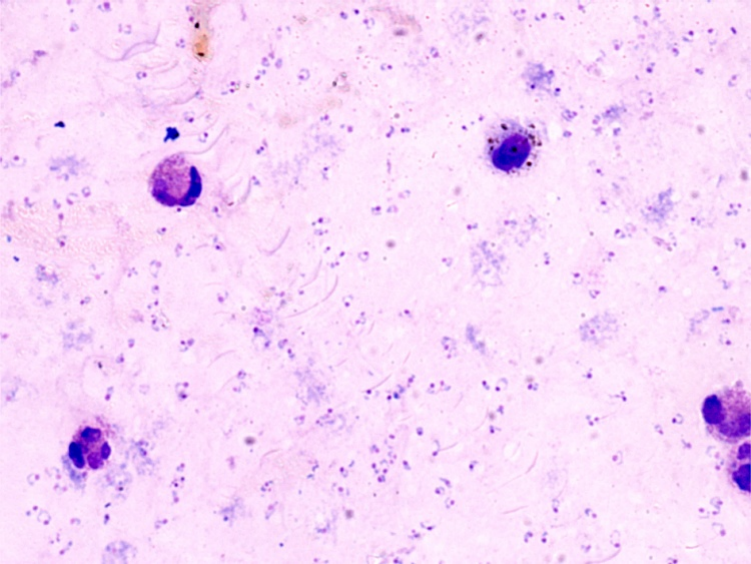
Giemsa stained thick film with high parasite density of *P. falciparum* (Photograph by Maria Gobbo, CTD Negrar)

Microscopy is still the gold standard for the diagnosis of malaria infection. The technique of slide preparation, staining and reading are well known and standardized, and so is the estimate of the parasite density, which is an added value of microscopy and that can easily be estimated on a thick film (**Figure 2**).^17^ RDTs are now replacing microscopy almost everywhere in endemic countries at P.H.C. level. A lot of different tests are available on the market and their accuracy is being systematically evaluated by the W.H.O. in partnership with other organizations.^27^,^28^ Several RDTs, both based on HRP-2 and on p-LDH, are virtually 100% sensitive at a comparatively low parasite density (200 parasites/μL), and also highly specific for *P. falciparum* malaria infection, some of them also for other plasmodia.

Other diagnostic methods do exist, such as the Quantitative Buffy Coat (QBC) and polymerase chain reaction (PCR), but they require adequate laboratory facilities and are not an option for routine use in endemic areas.

For practical purposes, RDTs are presently the main tool for the diagnosis of malaria in the field.

**Figure 3.**
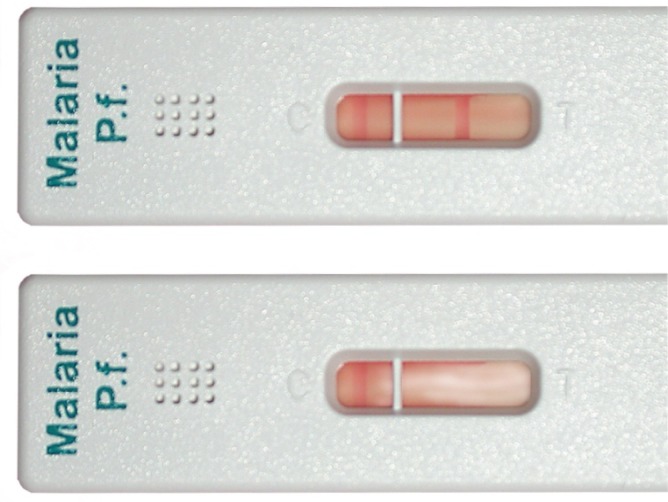
HRP-2 – based RDT with positive (a) and negative (b) result (Photograph by Maria Gobbo, CTD Negrar)

In immunochromatographic RDTs, malaria antigen is captured by monoclonal antibodies conjugated to a dye in a strip of nitrocellulose, causing a clearly visible line to appear. Most tests have a control line, that is the only one that appears in a negative test (**Figure 3, a**), while in the positive test a second line appears (**Figure 3, b**), usually within 15 minutes or less, making the reading straigthforward and reproducible, contrarily to microscopy. This is therefore an ideal tool in the field.

The systematic use of RDTs, now recommended by the WHO both for children and adults,^29^ should limit the prescription of antimalarials to those who really need it, thus diminishing the costs of malaria control programmes and preserving the efficacy of artemisinin-based drug combinations. Moreover, by excluding malaria as the cause of fever in RDT-negative subjects, health workers should take into account alternative causes of fever, also considering that in many countries malaria incidence has sharply declined and only a small proportion of all fevers are due to malaria.^30^–^33^

The new WHO policy has been preceded by a quite hot debate in the malaria community,^34^,^35^ and has been largely based on convincing results in countries where the decrease in malaria incidence had been dramatic, such as in Tanzania.^36^,^37^

The extension of this policy to countries where this decline has not yet occurred is questionable, though.

## Accuracy and cost effectiveness of RDTs for the diagnosis of malaria infection and of malaria-attributable fever

RDTs are an accurate tool for the diagnosis of malaria infection.^5^–^10^,^20^,^25^,^26^ Their sensitivity is sufficiently high to rule out malaria as the cause of fever in most instances, as they only miss very low parasite densities that are generally of no clinical significance. Nevertheless, in infants and young children the proportion of fevers attributable to malaria is very high even at the lowest parasite densities, that may be missed by RDTs.^20^,^38^ Moreover, in rare occasions, even a high or very high parasite density may be missed by a RDT, due to the so-called prozone effect^39^ or to other reasons. However, in general, refraining from malaria treatment in case of a negative RDT appears to be reasonably safe.^35^ Specificity is a more complex matter. It has been assessed by many studies on malaria infection and generally found to be very high, meaning that when a RDT result is positive, plasmodia are in fact present in the blood. However, contrarily to traditional microscopy, RDTs do not provide any estimate of the parasite density, and their specificity for malaria-attributable fever is highly variable, being very low in some epidemiological contexts.^20^ This means in practical terms that a patient with fever AND a positive RDT can have malaria, OR an alternative cause of fever. Prior to RDT introduction, clinical officers, who are most often nurses at the peripheral level of endemic countries, used diagnostic algorithms based on clinical symptoms and signs only, and they often treated a febrile patient with both an antimalarial and an antibiotic if malaria and a bacterial fever were both possible, according to their judgement. The use of RDTs should limit this “double prescription” by treating only with antimalarials those with a positive test, and with only antibiotics those who test negative for malaria, and have symptoms and signs indicating a possible bacterial cause.

While the latter option is probably reasonably safe, the former one is not. If a child with a fever due, say, to pneumonia is tested, and the RDT result is positive, the nurse may be suggested to treat only for malaria, while on clinical grounds he/she would have probably treated with both options.

As far as cost effectiveness is concerned, most studies have concluded that a RDT-based malaria management policy is cost-effective if compared to the presumptive management.^40^–^47^ However, a major flaw of most of these studies was the implicit assumption health workers would fully adhere to the (negative) test result, while at least in some countries it was clearly shown that a high proportion of febrile patients testing negative were nevertheless treated for malaria.^48^,^49^ Other researchers found more encouraging results, however in contexts where malaria incidence had dropped.^34^,^50^ Where and when malaria incidence is still high, such as in most of West Africa in the rainy season, a RDT-based policy was shown to be less cost-effective than the presumptive approach, and potentially harmful, especially for the possible consequences of a false positive result on the antibiotic prescription for non malarial febrile illnesses.^51^

A further limitation to cost-effectiveness of RDTs is the general tendency of using them on patients without fever, too,^48^,^52^ causing a waste of resources. Moreover, although we have seen that malaria infection without fever is not necessarily harmless, extending the use of antimalarials to afebrile patients would increase the risk of selection of drug resistant strains, that is exactly what the test-based policy should limit, instead.

## An evidence-based approach to malaria diagnosis and management

Malaria management policies cannot be the same everywhere, regardless the epidemiological context. Where malaria incidence is (or has become) low or very low, RDTs have clearly a key role in limiting unnecessary malaria prescriptions. Refraining from malaria treatment in such contexts is most probably safe.^34^,^35^ Health workers should however be properly trained on how to deal with the positive result: if the clinical picture is suggestive of a possible bacterial cause of fever, a double treatment (antimalarial plus antibiotic) is justified, as a positive RDT might be a false positive in clinical terms, by detecting malaria infection in a patient who actually has another acute disease. Clinical guidelines, including the Integrated Management of Childhood Ilnesses (IMCI), should introduce RDTs at the right step. The node on treatment decision for NMFI should come before the RDT node in clinical algorithms, and the RDT result should be used as a guide for malaria treatment, but not to exclude other potential causes of fever.^49^

In countries targeted for malaria elimination,^53^ RDTs and, potentially, new and even more sensitive diagnostic tools, may also be useful to identify pockets of infected people, regardless the presence of fever, and treat them, both to their benefit and that of the community.^54^

In contrast, in countries, or areas within countries, where malaria is still hyperendemic or holoendemic, the presumptive, clinical approach should be maintained, at least for children, and RDTs should not be used. For example, in a recent study in Burkina Faso during the rainy season, it was found that almost 90% of febrile children had a positive RDT.^20^ The device tested was Paracheck® Device, that is not one of the most sensitive RDTs according to W.H.O. assessment.^25^,^26^ With a more sensitive test the percentage of positive results would have probably approached 100%, making it totally useless as a decisional tool. It is clear that in such a situation using the test would only add the unnecessary costs of the test to that of the treatment, that would be administered to febrile children anyway.

## Diagnosing imported malaria in non endemic countries

What might be a correct approach for suspected imported malaria in a non endemic country? In this field of medicine, except for patients recently immigrating from holo-endemic countries, malaria infection equals malaria disease. Here unlimited diagnostic and therapeutical means are the rule. Comparison between different diagnostic tools is difficult: if microscopy remains the gold standard (reference test), the judgment will depend on how many fields have been examined. Currently more or less 100 high magnification fields are standard, but in some cases a diagnosis is made after careful scrutinizing a thick film during one or two hours, to find finally only one parasite! This, especially when *P.ovale* is at stake, or when a treatment, even a not specific one, e.g. with an antibiotic with antimalarial action such as cotrimoxazole, has already been started. PCR is highly sensitive, but will probably not outperform this lengthy microscopical search. A multi centre diagnostic study carried out by GISPI network in Italy confirmed that expert microscopy still remains the mainstay of the diagnosis of imported malaria, and this, particularly in mixed or in non falciparum infections, where it still outperforms alternative methods, including PCR.^55^

However, advanced diagnostic tools and expertise are not necessarily available in all care centers. In peripheral centers PCR is not available, and lab technicians lack expertise in microscopy. Moreover, malaria treatment should be initiated as soon as possible; diagnosis cannot wait until the next morning. As a consequence, RDTs have a substantial role in imported malaria: they are easy to perform, recent brands distinguish between falciparum and non falciparum, they have a sensitivity high enough to exclude a clinically important, imminent life threatening, falciparum infection. A further advantage is that a recent treatment does not necessarily hamper RDT sensitivity. National quality control of accuracy, compared to thick film simultaneously taken, is imperative since many caveats exist for correct use and interpretation.

## Conclusions and research needs

In an expert meeting at the European Commission DG research in 2010,^56^ experts were asked what the most relevant research needs on malaria diagnostics were. The basic conclusion was that we have already good tools for the diagnosis of malaria infection. However, in the context of malaria elimination programmes, research should focus on the development of even more sensitive tools, and ones that are able to detect also asymptomatic carriers (of gametocytes and not only asexual forms), thus contributing to detect pocket of infected individuals that can be successfully treated.^57^ For clinical management however, existing tools are already enough sensitive, as they are able to detect the vast majority of malaria-attributable fevers. The problem is the lack of specificity for clinical malaria, especially in high transmission settings. An ideal diagnostic tool would detect malaria infection as well as identify biomarkers of clinically significant malaria (capable of distinguishing disease from simple infection). Potential biomarkers have already been identified. In addition, an improved RDT for malaria would be able to give a semi-quantitative assessment of the parasite density. Alternatively, research should focus on incorporating in the same device markers of more than one disease, for example with the addition of a biomarker of bacterial disease to a malaria RDT.

One may wonder, though, if excessive reliance in new technology will not cause the definitive abandon of basic, microscopy-based laboratory in the field, that could still be an invaluable tool not only for the diagnosis of malaria, but also of TB, meningitis and other diseases, provided that adequate training, supervision and quality control are ensured.

## Figures and Tables

**Table 1.** Presence of fever according to parasitaemia in a population study.

	**fever**	**no fever**	**total**
**any parasite**	41	86	127
**no parasite**	33	247	280
**total**	74	333	407
